# Long-Term Outcomes of Mediterranean-Adapted Crohn’s Disease Exclusion Diet in Mild Pediatric Crohn’s Disease: A Real-Life Study from a Referral IBD Center

**DOI:** 10.3390/nu18081290

**Published:** 2026-04-20

**Authors:** Patrizia Alvisi, Maria Chiara Valerii, Enrico Perre, Gilda Barbieri, Fernando Rizzello, Marco Congiu, Arianna Pranzetti, Nikolas Kostantine Dussias, Francesca Sbravati, Veronica Imbesi, Enzo Spisni, Flavio Labriola

**Affiliations:** 1 Pediatric Gastroenterology Unit, Department of Paediatrics, Ospedale Maggiore, Azienda USL, 40133 Bologna, Italy; 2 Department of Biological, Geological and Environmental Sciences, Alma Mater Studiorum, University of Bologna, 40126 Bologna, Italyenzo.spisni@unibo.it (E.S.); 3 Specialty School of Pediatrics, Alma Mater Studiorum, University of Bologna, 40126 Bologna, Italy; 4 IBD Unit, IRCCS Azienda Ospedaliero-Universitaria of Bologna, 40138 Bologna, Italy; 5 Pediatric Unit, Department of Paediatrics, Ospedale Maggiore, Azienda USL, 40133 Bologna, Italy; marco.congiu@ausl.bologna.it

**Keywords:** pediatric Crohn’s disease, Mediterranean exclusion diet, exclusive enteral nutrition

## Abstract

**Background**: Exclusive enteral nutrition (EEN) is the recommended first-line therapy for induction of remission in pediatric mild-to-moderate Crohn’s disease (CD), but its restrictive nature often limits adherence and long-term sustainability. A modified version of the Crohn’s Disease Exclusion Diet (CDED), integrating Mediterranean dietary principles, was developed to offer a more acceptable alternative while preserving therapeutic efficacy. **Methods**: We conducted a retrospective, single-center study comparing short- and long-term outcomes of a Mediterranean-adapted CDED (M-CDED) with partial enteral nutrition (PEN) versus standard EEN in children with mild-to-moderate CD. Clinical remission was assessed after 8 and 16 weeks, while long-term outcomes were assessed after 1 and 2 years. **Results**: Data collected from thirty-two patients were analyzed (EEN, 14; M-CDED, 18). Clinical remission rates were comparable after 8 weeks (92.8% EEN vs. 94.4% M-CDED) and 16 weeks (100% in both groups). However, at 12 and 24 months, M-CDED was associated with significantly higher rates of clinical and biochemical remission and a markedly lower need for biologic drugs (12-month biologic initiation: 50% EEN vs. 11.1% M-CDED; *p* = 0.01). Adherence to M-CDED was excellent throughout follow-up. **Conclusions**: M-CDED with PEN appears to be as effective as EEN for remission induction, with improved long-term disease control and reduced therapeutic escalation. These findings support the feasibility of M-CDED as a sustainable option for long-term management of pediatric CD. Prospective studies are needed to confirm these results.

## 1. Introduction

Crohn’s disease (CD) is a multifactorial, chronic inflammatory disorder that can affect any part of the gastrointestinal tract, often in a discontinuous pattern. Together with ulcerative colitis (UC), the global incidence of pediatric inflammatory bowel disease (IBD) continues to rise, particularly in Northern Europe and North America, but also in Italy [1,2]. Although CD pathogenesis results from complex interactions among genetic, immunologic, and environmental factors, the rapid epidemiological increase suggests a major contribution from environmental influences, particularly dietary habits [3].

Nutritional therapy plays an essential role in the management of pediatric CD, both for induction of remission and for supporting growth and maintenance of adequate nutritional status. Western dietary patterns—high in processed foods, saturated fats, refined carbohydrates, and low in fiber, fruits, and vegetables—have been associated with dysbiosis, reduced microbial diversity, and loss of short-chain fatty acid-producing commensals [4,5,6,7,8,9]. These alterations impair epithelial barrier integrity and immune regulation, increasing intestinal permeability, promoting antigen translocation and chronic mucosal inflammation, and ultimately contributing to IBD onset and persistence [10,11,12]. Dysbiosis perpetuates intestinal barrier dysfunction and sustains mucosal inflammation [12,13]. Exclusive enteral nutrition (EEN) is currently recommended as first-line therapy for induction in mild-to-moderate pediatric CD [13,14,15]. EEN is associated with higher rates of clinical, endoscopic, and histologic healing, greater reductions in Pediatric Crohn’s Disease Activity Index (PCDAI) scores and with improved weight gain if compared with corticosteroid therapy [16]. However, EEN treatment often suffers from tolerability issues and poor compliance due to its exclusive reliance on liquid formulas and dietary monotony [16,17,18].

To overcome these barriers, the Crohn’s Disease Exclusion Diet (CDED) was developed to eliminate specific dietary components associated with mucosal inflammation, increased permeability and dysbiosis [18,19]. Randomized trials have demonstrated that CDED combined with partial enteral nutrition (PEN) is as effective as EEN for inducing remission, with better tolerance and good adherence [20,21]. However, the structure of the classic CDED remains challenging to follow, especially in the long term, requiring the consumption of liquid formulas and strict limitations on food choice [22,23,24,25,26]. Parents of children with CD experience significant stress related to maintaining specialized diets, which directly affects adherence [27]. Recently, new dietary models tailored to local food cultures have emerged as promising alternatives to standard nutritional therapies for Crohn’s disease [28]. Among these, the Mediterranean diet, rich in polyphenols, omega-3 fatty acids, fiber, and minimally processed foods, has gained increasing support in the literature as a beneficial approach in pediatric IBD, owing to its pleiotropic anti-inflammatory and microbiota-modulating effects [29,30,31]. Growing evidence suggests its efficacy in improving clinical symptoms, promoting clinical remission, and reducing inflammatory markers in pediatric IBD patients, while offering excellent safety, flexibility, and high patient and family acceptance [29,30,31,32,33,34].

EEN remains the recommended first-line therapy for induction of remission in pediatric CD, but its restrictive nature, limited acceptability, and the frequent disease recurrence following food reintroduction represent significant drawbacks in real-life settings. Moreover, evidence remains limited regarding optimal strategies for food reintroduction after EEN, as well as the ideal dosage and duration of partial enteral nutrition (PEN) when used beyond induction [33]. These limitations have prompted us to seek more sustainable dietary approaches that can maintain disease control while improving long-term adherence to nutritional therapy. Based on this rationale, we developed a modified dietary model grounded in the therapeutic principles of CDED and integrated with features of the Mediterranean diet (M-CDED), which is easier to follow over the long term and requires substantially lower PEN intake compared to the original CDED.

The aim of this study was to evaluate, in a real-world pediatric setting, the effectiveness of the Mediterranean-adapted Crohn’s Disease Exclusion Diet (in combination with partial enteral nutrition) in inducing and maintaining remission in children diagnosed with Crohn’s disease, and to compare its effectiveness with exclusive enteral nutrition (EEN). To this aim, we performed a retrospective analysis of clinical data collected from patients treated with EEN or with M-CDED and PEN, between September 2014 and July 2023.

## 2. Materials and Methods

### 2.1. Treatment Under Investigation

Two nutritional induction strategies were evaluated. The first strategy, exclusive enteral nutrition, consists of a polymeric formula (Modulen^®^, Nestlé, Vevey, Switzerland) administered as the sole source of calories for 8 weeks, in accordance with guidelines from European Crohn’s and Colitis Organisation and European Society for Paediatric Gastroenterology, Hepatology and Nutrition (ECCO–ESPGHAN) [35,36,37]. This phase is followed by a gradual reintroduction of solid foods over 2 weeks and subsequent transition to a free diet. The second strategy was the Mediterranean-adapted Crohn’s Disease Exclusion Diet (M-CDED) combined with partial enteral nutrition (PEN). This diet was administered for a total of 16 weeks and was followed by a maintenance phase. This second dietary approach integrates the principles of the traditional CDED with Mediterranean dietary features, as detailed below.

### 2.2. Mediterranean-Adapted Exclusion Diet (M-CDED)

The CDED protocol associated with PEN was developed by Arie Levine and collaborators [22] to induce remission in children with mild to moderate CD, with better tolerability than EEN. CDED is a structured, food-based regimen developed to reduce exposure to dietary components that may promote intestinal inflammation and dysbiosis. It systematically excludes ultra-processed foods, additives such as emulsifiers and maltodextrin, and emphasizes minimally processed foods, fruits, and vegetables, combined initially with PEN. Using the same core principles of an exclusion diet with greater tolerability and flexibility, our nutrition team developed a modification of the original CDED according to Mediterranean dietary principles (M-CDED). This diet involves several steps in which PEN is progressively reduced until it is abandoned and most of the typical foods of the Mediterranean diet are reintroduced, making it easier for children and their families to use this diet as a sustainable healthy dietary pattern. This dietary protocol consists of a 16-week induction protocol, divided into two progressive 8-week phases (Phase 1 and Phase 2), each further divided into two four-week periods, followed by a maintenance diet (Phase 3) extended as long as possible.

During Phase 1a (weeks 1–4), patients receive approximately 50% of their estimated daily caloric needs, based on the latest recommendations of the Italian Dietary Guidelines (IDGs) [38,39,40], administered as a 20% reconstituted polymeric formula (1 kcal/mL). For the rest of the caloric intake, allowed foods in this phase mirror the classic CDED, with two notable differences: a moderate amount of high-quality aged cheeses was included to ensure adequate calcium intake, and seasonal fruits and vegetables rich in polyphenols and flavonoids with potential anti-inflammatory effects were incorporated.

During Phase 1b (weeks 5–8), PEN contribution is reduced to 25% of total caloric intake, while permitted foods remain unchanged from Phase 1a.

Phase 2a (weeks 9–12) involves a further reduction in PEN to 15%. A gradual reintroduction of gluten-containing foods is achieved through the introduction of ancient-grain flours, due to their lower inflammatory impact [41]. Also, legumes are reintroduced but only as peeled or pureed, to reduce the FODMAP content. Legumes are reintroduced to address the common iron deficiencies observed in patients with CD [42]. The types of fruit and vegetables allowed are gradually increased.

From weeks 13–16 (Phase 2b), PEN is discontinued and foods rich in calcium are reintroduced using non-bovine cheeses to reduce intake of the pro-inflammatory Casein A1 protein [43]. Throughout all phases, consumption of processed meats, refined sugars, ultra-processed products, and sugar-sweetened beverages are strongly discouraged.

From week 17 onward, patients follow a maintenance regimen allowing two flexible meals per week to promote long-term adherence. Allowed and excluded foods in the M-CDED are described in Table 1.

Unlike the original CDED, the M-CDED provides greater flexibility from the early stages. While daily intake of certain foods (e.g., animal protein sources and pectin-rich fruits) is recommended, these can be adjusted based on individual tolerance and nutritional needs. Regular consumption of fruits and vegetables remains a core target, with patients personalizing the diet under dietitian supervision.

To support adherence, all patients receive educational materials—including recipe booklets and sample meal plans adapted to local food preferences—at each phase transition.

### 2.3. Study Design and Patients

**Figure 1:**
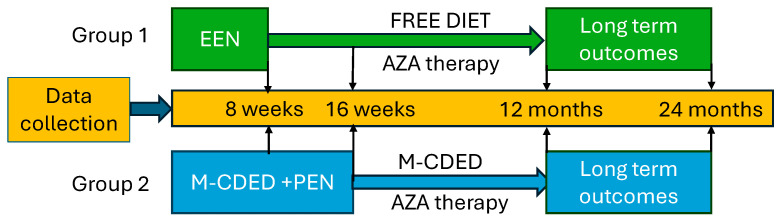
M-CDED retrospective study design. AZA, Azathioprine.

We conducted a retrospective, single-center analysis of pediatric patients diagnosed with mild-to-moderate CD, defined as patients with Pediatric Crohn’s Disease Activity Index (PCDAI) < 30 [44,45]. We compared a cohort treated with EEN (Group 1) to one treated with M-CDED + PEN (Group 2). We first analyzed the efficacy of these two treatments in inducing remission (8 weeks and 16 weeks after the protocol started) and then the long-term outcomes of the disease (after 12 and 24 months from the beginning of the therapies). After the end of nutritional protocols (8 weeks for EEN and 16 weeks for M-CDED), all patients started azathioprine therapy at a standard dose (2 mg/kg/day) as the only pharmacological maintenance therapy. Patients in the M-CDED group continued the dietary intervention, whereas patients in the EEN group transitioned to a free diet. The study design is shown in Figure 1. All patients were treated between September 2014 and July 2023 at the Pediatric Gastroenterology Unit of the Maggiore Hospital in Bologna, Italy. CD was diagnosed according to the Porto criteria [46].

Eligibility criteria were defined to ensure clinical homogeneity and adequate data completeness for retrospective analysis.

Inclusion criteria were: age < 18 years at diagnosis, regardless of age at the time of data collection; diagnosis of mild-to-moderate CD with a non-stricturing, non-penetrating phenotype (B1, Paris classification) [45]; initiation of nutritional therapy or EEN as first-line induction treatment; availability of at least 12 months of follow-up; availability of completed dietary adherence questionnaires during the observational window in patients treated with M-CDED. Patients were included only if sufficient clinical, laboratory, and follow-up data were available in the medical records to allow reliable outcome assessment. For outcome analysis in this retrospective study, visits were considered eligible only if they occurred within ±1 week of the scheduled primary outcome assessment and within ±1 month of the 12- and 24-month follow-up timepoints.

Exclusion criteria were: severe or complicated CD (B2/B3 phenotypes); presence of perianal disease; active extraintestinal manifestations; any previous intestinal surgery. All enrolled patients were routinely screened and resulted negative for Celiac Disease and gluten sensitivity.

### 2.4. Outcomes

The primary outcomes were clinical remission, defined as a PCDAI score < 10, and biochemical remission, defined as FC < 250 μg/g and CRP < 0.5 mg/dL.

Secondary outcomes included disease progression, defined as the need for escalation to biological therapy or surgery during follow-up, and changes in anthropometric parameters (weight, height, and body mass index).

### 2.5. Data Sources and Data Extraction

All data were retrospectively extracted from routinely collected clinical records, including electronic medical charts and standardized documentation used in daily clinical practice at the study center.

Collected variables included demographic data, disease characteristics according to the Paris classification, PCDAI scores, laboratory inflammatory markers (CRP and FC), hemoglobin, anthropometric measurements, and information on concomitant therapies.

Dietary adherence data were obtained from structured nutritional interviews in the EEN group and from the Modified Crohn’s Disease Exclusion Diet Adherence Report Scale (MARS) [45] questionnaires in the M-CDED group, routinely administered during outpatients’ visits. The questionnaire includes 11 items, with a total score of 13 points (provided in Supplementary Table S1). A score > 10 indicated good compliance, 7–10 moderate compliance, and <6 poor/non-compliance [47,48]. Data were recorded in an anonymized Excel database (Microsoft Corporation, Redmond, WA, USA).

### 2.6. Timing of Assessments

For outcome analyses in this retrospective study, visits were considered eligible only if they occurred within predefined time windows.

Primary outcome assessments were based on visits occurring at 8 and 16 weeks from treatment initiation, with an acceptable window of ±1 week.

Long-term outcomes were assessed using visits occurring at 12 and 24 months from treatment initiation, with an acceptable window of ±1 month.

### 2.7. Statistical Analysis

Data were analyzed using JASP software (version 0.18.3, 2024; JASP Team, Amsterdam University, Netherlands.). Continuous variables were expressed as median and interquartile range (IQR) or mean and standard deviation (SD), according to their distribution. Categorical variables were presented as counts and relative frequencies. Comparisons between two groups of continuous variables were performed using the Wilcoxon–Mann–Whitney test or Student’s T-test, as appropriate. Comparisons among more than two groups were conducted using ANOVA, followed by Bonferroni’s post hoc test for pairwise comparisons, or the Kruskal–Wallis test followed by Dunn’s post hoc test for nonparametric data. Categorical variables were analyzed using the Chi-square test. Associations between treatment groups (Group 1 vs. Group 2) and categorical outcomes (clinical remission, biochemical remission, need for biologics, and need for surgery) were evaluated using Fisher’s exact test, given the presence of small cell counts. *p* values < 0.05 and <0.01 were considered statistically significant and highly significant, respectively.

### 2.8. Ethical Issues

All participants were fully informed about the study and provided informed consent to participate. Written consent was obtained from each participant (in the case of children, from one parent; for adolescents, from both a parent and the adolescent). The study was approved by the CE-AVEC Ethics Committee (protocol no. 0142896-2/12/24).

## 3. Results

### 3.1. Baseline Characteristics

Sixty-nine patients were screened, and thirty-two met the inclusion criteria: 14 were treated with EEN (Group 1; mean age, 12.6 ± 2.8 years) and 18 with M-CDED (Group 2; mean age, 13.2 ± 3.5 years). The median time from diagnosis to initiation of nutritional therapy was 1 month (IQR, 0.1–2 months). Baseline characteristics—including sex, disease location, Paris classification, and disease severity (as evaluated by PCDAI and endoscopic findings)—did not differ significantly between groups and are summarized in Table 2.

### 3.2. Short-Term Outcomes

**Figure 2:**
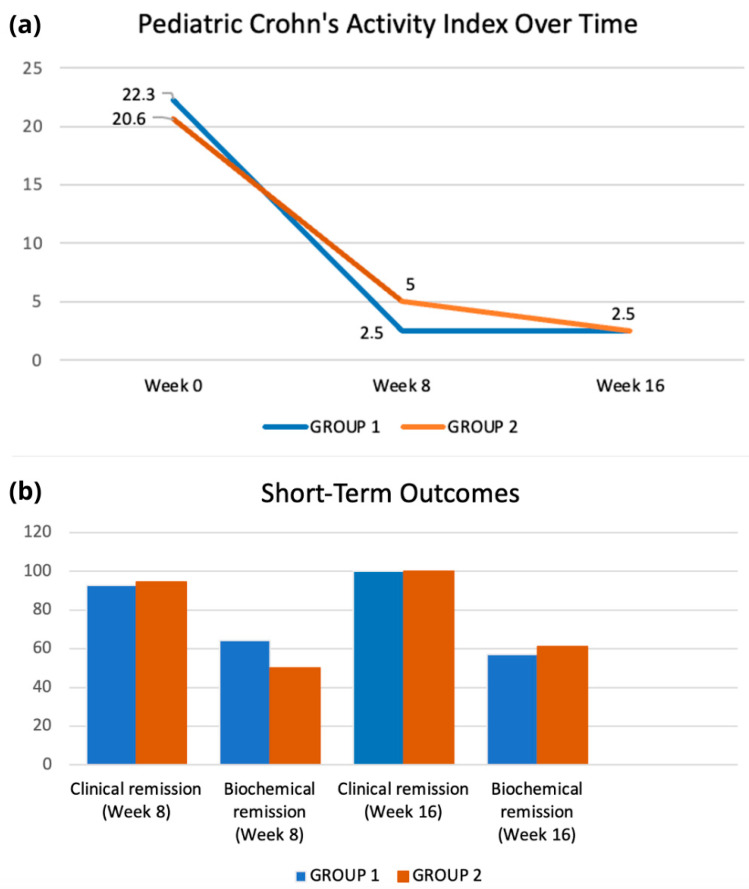
PCDAI and laboratory short-term outcomes. (**a**) PCDAI median values over a 16-week period in patients treated with EEN (Group 1) or M-CDED (Group 2). (**b**) Percentage (%) of patients achieving clinical and biochemical remission after 8 and 16 weeks of intervention. No difference between the two groups showed statistical significance at *p* < 0.05.

Short-term outcomes after 8 and 16 weeks of nutritional treatment are shown in Figure 2 and Table 3. Regarding clinical remission, no statistically significant differences were observed between the M-CDED and EEN groups at either 8 or 16 weeks of treatment. At week 8, clinical remission was achieved in 13/14 patients (92.8%) in Group 1 (EEN) and in 17/18 patients (94.4%) in Group 2 (M-CDED) (*p* = 0.85). At week 16, clinical remission was sustained in 100% of patients in both groups. Biochemical remission occurred in 9/14 patients (64.3%) in Group 1 (EEN) and in 9/18 patients (50%) in Group 2 (M-CDED) (*p* = 0.42). At week 16, biochemical remission rates remained comparable between Group 1 (EEN, 8/14 patients, 57.1%) and Group 2 (M-CDED, 11/18 patients, 61.1%) (*p* = 0.77).

As shown in Table 3, a significant decrease in CRP and FC values occurred over the 16-week treatment period in both groups compared to baseline. After 16 weeks, only CRP values showed statistically significant differences between the two groups, although this finding is largely explained by the different baseline CRP values.

#### Changes in Anthropometric Parameters During the Induction Phase

Anthropometric parameters are detailed in Supplementary Table S2. During the 16-week period, all patients in both groups showed a mild increase in body weight and BMI, with no significant differences observed between the two groups.

### 3.3. Long-Term Outcomes

**Figure 3:**
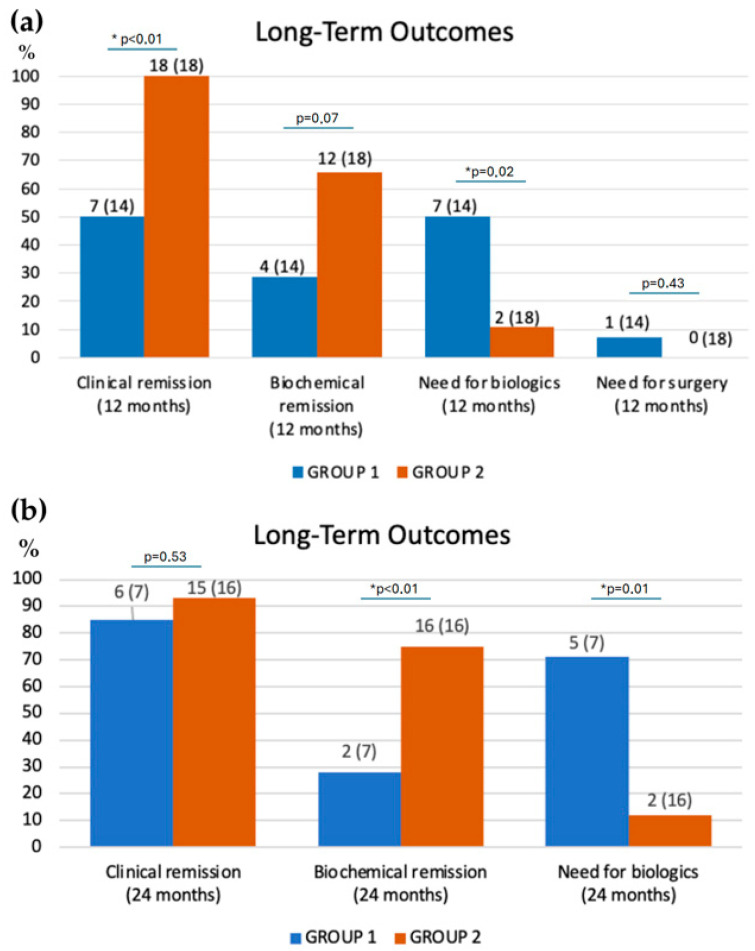
Long-term outcomes in patients treated with EEN (Group 1) or M-CDED (Group 2). (**a**) Number (above the total number) of patients achieving clinical and biochemical remission, or who required biologic or surgical therapy after 12 months of follow-up from start of nutritional treatment. (**b**) Remaining patients achieving clinical and biochemical remission, or who required biologic therapy after 24 months of follow-up from the start of nutritional treatment. * Indicates a statistically significant difference between the two groups; *p* values are showed.

Long-term outcomes after 12 and 24 months of nutritional and pharmacologic treatment in both groups are shown in Figure 3. After 12 months, clinical remission rate significantly increased in Group 2 (100% vs. 50%, *p* < 0.01) while biochemical remission rate only reached a *p* value close to statistically significant, that defines a trend considering the low number of subjects. (66% vs. 29%, *p* = 0.07). Moreover, biologic therapy was required in only 2 of 18 patients (11%) in Group 2, compared with 7 of 14 (50%) in Group 1 and this difference was statistically significant (*p* = 0.02). Only one patient from Group 1 required surgical treatment for Crohn’s disease, whereas no one did in Group 2, with no statistically significant difference.

Clinical data were collected at 24 months, from the remaining patients, since children undergoing biological therapy or surgery during the first year were excluded from the second-year clinical data collection. This is because the drug used (anti-TNF-α) or surgery could significantly affect both clinical and biochemical remissions. For this reason, at 24 months the number of participants was reduced, particularly in Group 1 in which, during the first 12 months of follow-up, eight children (only two in Group 2) showed worsening of the disease which required the initiation of biological therapy or surgery.

After 24 months, clinical remission rates showed no statistically significant difference between groups: 15 of 16 (94%) in Group 2 versus 6 of 7 (86%) in Group 1 (*p* = 0.53). However, biochemical remission rates were still statistically different, with 16 of 16 (100%) patients in Group 2 achieving biochemical remission and only 2 of 7 (29%) in Group 1 (*p* < 0.01). After 24 months, the proportion of patients requiring biologic therapy remained stable at 2 of 16 (12%) in Group 2, while it increased to 5 of 7 (71%) in Group 1 (*p* = 0.01).

Overall, considering 2 years of follow-up, only 4 of 18 patients (22.2%) needed to initiate biological therapy with anti-TNF-α in Group 2 (M-CDED), while 12 of 18 patients (67%) initiated anti-TNF-α therapy in Group 1 (EEN and then free diet). This difference is considered highly significant, with *p* < 0.01.

### 3.4. Adherence to M-CDED

**Figure 4:**
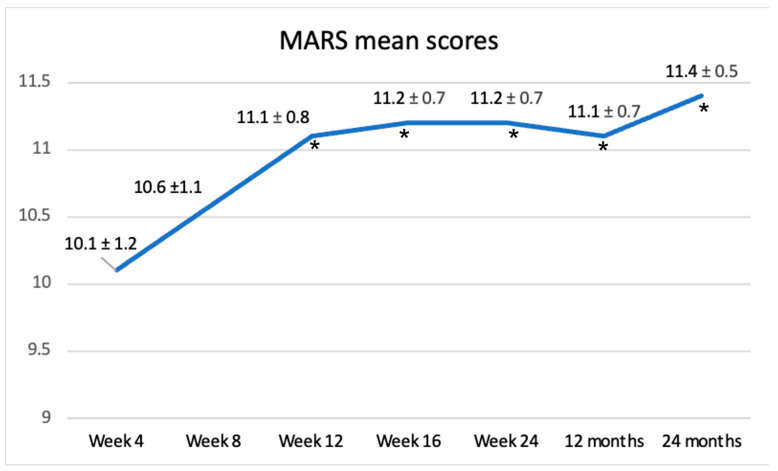
Adherence to M-CDED evaluated using MARS scores. Mean scores and SD calculated using the MARS questionnaire, recorded every 4 weeks from week 4 to week 24, then after 12 and 24 months from the beginning of intervention. * Indicates a statistically significant difference at *p* < 0.05 compared to the adherence value observed after 4 weeks.

Regarding adherence to M-CDED, all 18 patients (100%) in Group 2 who completed the 16-week treatment fully adhered to the study protocol up to 24 months, even when biologic therapy was required. A complete adherence was obtained in all 14 patients (100%) in Group 1 at week 8. In Group 2, mean MARS scores (showed in Figure 4) indicated good adherence, with progressively increasing values up to week 24 (11.2 ± 0.6). Among the patients interviewed at 24 months, the mean MARS score confirmed sustained good adherence (11.4 ± 0.5), indicating that the M-CDED can be easily integrated into the lifestyle of these patients.

## 4. Discussion

This single-center, retrospective study compared the short- and long-term effectiveness of a Mediterranean-adapted CDED (M-CDED) in a cohort of pediatric patients with Crohn’s disease. In this real-life study, M-CDED plus PEN demonstrated comparable short-term efficacy compared to EEN, with similar clinical and biochemical remission rates after 8 and 16 weeks. Long-term outcomes strongly favored M-CDED with significantly superior rates of clinical and biochemical remission, as well as a marked reduction in the need for biologic therapy.

Both induction strategies achieved comparable clinical remission rates after 8 weeks and 16 weeks (100% in both groups) with no significant differences in clinical or biochemical remission. These clinical findings support the similar short-term efficacy of structured exclusion-based diets, including both the conventional CDED and our Mediterranean-adapted version, for short-term induction of remission in mild-to-moderate pediatric Crohn’s disease. In line with this evidence, Levine et al. demonstrated similar or better induction remission rates with CDED plus PEN versus EEN at 6 and 12 weeks, along with higher corticosteroid-free remission and better adherence [22].

Our data are consistent with this evidence and subsequent studies, suggesting that structured exclusion diets can provide an effective alternative to formula-only treatment without compromising short-term outcomes [49,50,51,52].

Biochemical remission rates were also similar at week 16 between the two groups, although baseline CRP was higher in Group 2. In fact, only in group 2 the difference between baseline and 16 weeks was statistically significant. Fecal Calprotectin (FC) showed a slight increase at week 16 in the EEN group, while values continued to decline in the M-CDED cohort, although without reaching statistically significant differences. This pattern aligns with previous studies showing that CDED-based approaches may lead to more sustained reductions in inflammatory markers compared with EEN, through a gradual and structured reintroduction of foods [22,23,50,51,52]. In Levine’s study, after 12 weeks, CRP normalization was achieved in 75.9% of patients in the group treated with CDED combined with PEN, compared with 47.6% in the EEN group, with no statistically significant difference between the two groups [22]. Although current ECCO–ESPGHAN guidelines do not formally recommend CDED as maintenance therapy due to limited long-term evidence, emerging pediatric studies increasingly support its role in sustaining remission [53]. The multicenter DIETOMICS randomized trial has reinforced the emerging role of CDED-based nutritional strategies in sustaining remission beyond induction. A structured exclusion diet may achieve durable disease control and mucosal improvement even beyond the induction phase, with higher adherence and better nutritional outcomes compared to the traditional EEN approach. Particularly relevant is the observation that 90% of patients on CDED monotherapy (without immunomodulators) maintained remission at week 14, suggesting that in a selected subset of adherent patients, CDED could also function as a standalone maintenance therapy [54]. Levine and collaborators also demonstrated how patients who transitioned from EEN back to a free diet tended to develop intestinal dysbiosis, characterized by an increase in pro-inflammatory bacterial species such as Proteobacteria. On the other hand, CDED helped maintain a healthier microbial balance, marked by an increase in beneficial bacteria like *Faecalibacterium prausnitzii* and a decline in harmful strains like *Escherichia coli*. This shift in microbiota composition also promoted the production of butyrate, a short-chain fatty acid with strong anti-inflammatory properties that also support intestinal barrier integrity [22,23]. Despite its great potential, traditional CDED protocols can be perceived as restrictive and difficult to maintain, due to limited food variety and high reliance on formula [24,25]. For example, while many patients reported the diet to be feasible at home, fewer found it feasible in work or school settings [47,48]. Beyond CDED, the Mediterranean diet—rich in fruits, vegetables, whole grains, and healthy fats—has also been recognized for its anti-inflammatory effects in IBD, including CD [55,56]. Both of these diets are capable of modulating the intestinal microbiota and microbial metabolites with a therapeutic role in IBD [57,58]. Integrating anti-inflammatory-focused diets such as CDED and the Mediterranean may therefore represent a promising, though still evolving, adjunct to pharmacological therapies in managing Crohn’s disease. However, while microbiota modulatory effects are well documented in adult populations and rodent models, conclusive data on the Mediterranean diet in pediatric Crohn’s disease remain speculative [59,60].

The introduction of the M-CDED into clinical practice aimed to retain the therapeutic benefits of the CDED while integrating a Mediterranean dietary pattern and a multiphase approach designed to be gradually less restrictive and therefore more feasible for long-term adherence.

Based on our data, M-CDED demonstrated its most notable advantages in the long-term follow-up. At both 12 and 24 months, significantly higher rates of clinical and biochemical remissions were observed in the M-CDED group with respect to the “free diet” ones. Moreover, although both groups received azathioprine maintenance therapy, the need for escalation to biologic therapy was substantially lower in the M-CDED cohort at 12 and 24 months, suggesting that dietary therapy may exert a medication-sparing effect in selected adherent individuals. Finally, our study showed excellent adherence to M-CDED, maintained at 12 and 24 months, as assessed by the MARS questionnaire.

Recent clinical studies indicate that eating disorders, particularly avoidant/restrictive food intake disorder (ARFID), are relatively common in individuals with IBD, with screening-based prevalence ranging from approximately 10% to 18% of patients (including pediatric and young adult populations) and higher rates of disordered eating behaviors reported compared with general populations [61,62,63,64,65,66]. Disease activity, frequent relapses, and low adherence to healthy eating patterns represent significant risk factors for the development of eating disorders in children and adolescents with IBD, therefore increasing the possibility of inadequate intake of energy, macronutrients and micronutrients [66,67]. The absence of any eating disorder onset within our cohort during the observation period supports the view that a structured, multidisciplinary team approach—involving gastroenterologists, dietitians, psychologists—is crucial for early screening, prevention and management of eating disorders in this fragile population [67,68]. A varied diet that aligns with individuals’ cultural eating habits, such as the Mediterranean diet for our population, could support adequate nutritional intake, good adherence and enhanced psychological well-being [65].

We are aware that the single-center retrospective design and the small sample size limit the statistical significance of our analysis. However, we must remember that Crohn’s disease is a rare condition in pediatrics that affects less than 10 children out of 100,000 in Southern Europe [69]. For this reason, most of the published clinical studies regarding pediatric CD do not exceed thirty patients [70,71,72]. Endoscopic evaluation was not systematically performed in our center during follow-up, to limit as much as possible invasive procedures on children. Although in other studies endoscopy procedures were routinely repeated after 6 months [28], we prefer to measure FC levels since they statistically correlate with the presence of mucosal lesions [73]. After diagnosis, we performed subsequent endoscopies only after flare-up of the disease, before starting biological anti-TNF-α therapies. From the baseline to the end of the observations, we did not observe significant changes in Hb values, which remained unchanged in both groups. Additionally, due to the observational nature of the study, gut microbiota composition and microbiome metabolites, such as short-chain fatty acids, were not analyzed in these cohorts since ESPGHAN guidelines do not suggest microbiota and/or microbiome analyses as parameters usable in clinical practice. Despite these limitations, this study provides relevant real-world evidence regarding the feasibility and potential long-term benefits of an innovative M-CDED in pediatric CD. Overall, this study highlights the M-CDED as a potentially more sustainable and acceptable approach for the management of pediatric Crohn’s disease.

## 5. Conclusions

M-CDED appears to be a feasible and well-tolerated alternative to EEN for both induction and maintenance therapy in pediatric patients with mild-to-moderate Crohn’s disease.

The integration of Mediterranean dietary principles with exclusion diet concepts may provide a sustainable and patient-friendly nutritional strategy for chronic disease management, even in the absence of standardized post-induction guidelines. Future multicenter, prospective studies should incorporate endoscopic endpoints, longer follow-up durations, microbiome analyses and systematic evaluation of maintenance approaches to better define the role of M-CDED in pediatric IBD management.

## Figures and Tables

**Table 1:** Allowed and excluded foods in the M-CDED. Each phase outlines specific examples of included or gradually reintroduced foods, as well as those that remain excluded.

Phase	Allowed Foods
Phase 1	Cereals (1 serving per day) White rice (Basmati, Jasmine, or parboiled—not brown rice), rice pasta or noodles (only once per day, without added cereals, additives, emulsifiers, or preservatives), and rice flour. Proteins Organic chicken breast and organic eggs (regularly), fresh fish (once a week) such as sea bream, cod, hake, sea bass, or sole, and hard cheese like Parmigiano Reggiano DOP or aged pecorino (1 teaspoon per day). Fruits (3 servings per day) Apple (peeled, not quince), banana and others depending from season: during spring and summer, allowed fruits are strawberries, melon, watermelon, apricots, peeled peaches, while in autumn and winter: a glass of freshly squeezed orange juice per day, along with allowed fruit and vegetable juices or extracts. Vegetables (1 serving of potatoes + 1 of others per day) Whether fresh or frozen, should include one serving of potatoes and one serving of other allowed vegetables between lunch and dinner (approximately 50 g raw or 100–150 g cooked). Seasonal vegetables are recommended. Spring–Summer: peeled (cooled) potatoes/sweet potatoes, steamed carrots, spinach/beet greens (no stalks), lamb’s lettuce, zucchini, asparagus tips. Autumn–Winter: same, excluding zucchini and asparagus. Condiments Extra virgin olive oil, iodized salt (moderate), fresh herbs (rosemary, basil, mint, thyme, etc.), minimal fresh garlic/onion (for sautéing), lemon juice (fresh only). Moderate honey and unrefined cane sugar (max 2–3 tsp/day). Drinks Still water (room temp preferred), tea, chamomile, bagged herbal infusions (not powdered). Optional addition: lemon, lime, orange slices, or mint. Cooking methods Grilling, baking, steaming, boiling, or non-stick pan with minimal oil. Frying is not allowed.
Phase 2	All foods allowed in Phase 1 with newly introduced foods. Cereals Pseudocereals (buckwheat, amaranth, millet, quinoa, sorghum), pasta (corn/buckwheat-based), (chickpeas, peas, lentils), couscous (corn or buckwheat), others (corn flour, rolled oats). Proteins Fresh oily fish (sardines, anchovies, mackerel, herrings), yogurt (plain cow or goat, Greek, or kefir without added sugars or thickeners), skinned or pureed legumes. Fruits Seasonal fruits: cherries, plums, grapes, peeled almonds, walnuts. Vegetables Cucumbers, eggplants, peeled bell peppers, tomatoes, radishes, agretti (saltwort), green radicchio, turnips. Other Dark chocolate (maximum 2 squares/day). Cooking methods Frying allowed up to once per week.
Phase 3	Cereals Brown rice, bread and pasta and cous cous made from ancient grains (kamut, farro) or legume based. Naturally gluten-free flours (e.g., oat, legume, almond) as well as flours made from ancient grains, permitted in 1 portion/day for homemade sweet/savory recipes. Proteins Organic white meats (e.g., turkey, rabbit) maximum twice a week, and lean, unprocessed organic red meat, allowed once per week. The range of fish now includes all fresh varieties, with oily fish still recommended once weekly and the occasional inclusion of shellfish or canned fish if tolerated. The variety of cheese now includes aged goat cheese, naturally lactose-free cheeses (e.g., aged pecorino, lactose-free ricotta, also made from goat whey), free from additives and thickeners. Legumes are further expanded to beans, fresh or frozen legumes, and eventually whole dried legumes. Properly rinsed canned legumes are also allowed, as long as they contain only legumes, water, and salt. Fruits Pears, quince, persimmons, figs, prickly pears, berries, pomegranate, chestnuts, loquats, and avocado. Vegetables Pumpkin, artichokes, all varieties of cabbage, broccoli, lettuce, celery, mushrooms, chicory, leeks, and red radicchio. Potato or sweet potato consumption is now reduced to 1–2 times per week. Condiments Oilseeds, particularly flaxseeds and sesame seeds, are also allowed and can be included regularly. Other Daily plant-based options now include nuts (e.g., pistachios, pine nuts, hazel nuts) while continuing to favor walnuts.
Excluded	Processed meats (such as hot dogs, sausages, canned meat, bacon, and similar products), except for dry-cured ham aged at least 24 months and free from nitrites or nitrates, which may be consumed up to once a week. Sugary and carbonated beverages. Frozen or ready-made products, such as frozen pizza, packaged puffs or shortcrust pastry, and pre-packaged flatbreads. Foods from fast-food and with high content of processed sauces. Sweets, such as homemade cakes or ice cream, are allowed only on weekends, limited to one serving per day.

**Table 2:** Baseline characteristics of patients treated with EEN (Group 1) or M-CDED (Group 2). Disease location is defined by Paris Classification: L1 (terminal ileum), L2 (colon), L3 (ileocolonic), or L4 (upper GI).

	Group 1 (*n* = 14)	Group 2 (*n* = 18)	*p*-Value
Age at diagnosis (mean, SD)	12.6 ± 2.8	13.2 ± 3.5	0.63
Male, *n* (%)	10 (71.4)	11 (61.1)	0.54
A1a (<10 years) (*n*, %)	1 (7.1)	0	0.25
PCDAI	22.3 ± 3.8	20.6 ± 5.7	0.34
Location			
L1—ileal/ileocecal, *n* (%)	4 (28.6)	6 (35.3)	0.69
L2—colonic, *n* (%)	1 (7.1)	0	0.26
L3—ileocolonic, *n* (%)	8 (57.1)	11 (64.7)	0.67
L4—upper GI, *n* (%)	5 (35.7)	7 (38.8)	0.61

**Table 3:** Changes in the mean (±SD) and median (IQR) values of the laboratory parameters 8 and 16 weeks after nutritional therapy in patients treated with EEN (Group 1) or M-CDED (Group 2). *p* values are calculated in comparison with the baseline values, while the final column represents the *p*-values obtained in the comparison between the two groups. Hb = Hemoglobin; CRP = C-Reactive Protein, FC = Fecal Calprotectin.

	Group 1 (*n* = 14)				Group 2 (*n* = 18)				Group 1 vs. Group 2
Parameter	Baseline	Week 8	Week 16	*p*-value	Baseline	Week 8	Week 16	*p*-value	*p*-value
Hb (g/dL, mean, SD)	12.4 ± 1.8	12.7 ± 1.5	12.7 ± 1.4	0.81	12.2 ± 1	12.7 ± 1.3	12.4 ± 1.6	0.64	0.55
CRP (mg/dL, median, IQR)	0.2 (0.2–0.7)	0.1 (0–0.2)	0.1 (0.1–0.4)	0.01	1.2 (0.9–5.7)	0.3 (0.1–0.6)	0.5 (0.1–1.4)	<0.01	0.03
FC (μg/g, median, IQR)	508 (203–1096)	150 (40–800)	100 (45–279)	0.02	550 (298–795)	87 (40–204)	172 (59–318)	<0.01	0.78

## Data Availability

The data presented in this study are available on request from the corresponding author due to privacy and legal reasons.

## Supplementary Materials

The following supporting information can be downloaded at: https://www.mdpi.com/article/10.3390/nu18081290/s1, Table S1: Modified Modulen/CDED Adherence Report Scale (MARS); Table S2: Anthropometric parameters.

## Abbreviations

The following abbreviations are used in this manuscript:

EEN	exclusive enteral nutrition
CD	Crohn’s disease
M-CDED	Mediterranean-adapted Crohn’s Disease Exclusion Diet
PEN	partial enteral nutrition
CRP	C-Reactive Protein
FC	Fecal Calprotectin
PCDAI	Pediatric Crohn’s Disease Activity Index
MARS	Modified Modulen/CDED Adherence Report Scale
BMI	body mass index
