# Supplementary material for: Long-Term Outcomes of Mediterranean-Adapted Crohn’s Disease Exclusion Diet in Mild Pediatric Crohn’s Disease: A Real-Life Study from a Referral IBD Center
Source: Nutrients. 2026 Apr 20;18(8):1290. doi: 10.3390/nu18081290 (PMC13119448; doi:10.3390/nu18081290)
Supplement: Supplementary file 1 [file nutrients-18-01290-s001.zip › nutrients-4195683-supplementary.pdf]

SUPPLEMENTARY MATERIALS

Supplementary Table S1 - Modified Modulen/CDED Adherence Report Scale (MARS)

		Never	Rarely	Sometimes	Often	Very often	Always
**1.**	**Diet instructions followed as prescribed the last 3 weeks**	0	0	1	1	2	2
		**Yes**	**No**				
**2.**	**Followed other diet than explained**	0	1				
**3.**	**Forgot to follow the diet instructions**	0	1				
**4.**	**Temporarily stopped the diet instructions**	0	1				
**5.**	**Only followed the diet instructions when active symptoms**	0	1				
**6.**	**Ignored the diet for a meal**	0	1				
**7.**	**Sometimes used not allowed ingredients**	0	1				
**8.**	**Used not allowed ingredients regularly**	0	1				
**9.**	**If I could, I would avoid the diet instructions**	0	1				
**10.**	**I followed diet instructions daily**	1	0				
		**Yes**	**No, used more**	**No, used less**			
**11.**	**Used Modulen IBD® as advised**	1	2	0			

[1S] MARS questionnaire describes patient compliance with dietary therapy: > 10 points = Good compliant, 7-10 points = Moderately compliant, < 6 points = Poor/Non-compliant.

Supplementary Table S2 - Anthropometric Parameters

	Group 1 (n=14)	Group 2 (n=18)	p-value
**Anthropometric parameters at screening**			
**Weight (Kg, SD)**	40.4 ± 10	41.8 ± 12	0.73
**Height (cm, SD)**	154.1 ± 17.4	151.7 ± 16	0.7
**BMI (Kg/m², SD)**	16.8 ± 2	17.7 ± 2.5	0.27
**Anthropometric parameters at week 8**			
**Weight (Kg, SD)**	43.4 ± 9.8	42.7 ± 12.4	0.85
**Height (cm, SD)**	154.9 ± 16.2	152.3 ± 16	0.65
**BMI (Kg/m², SD)**	17.8 ± 1.4	18 ± 2.7	0.89
**Anthropometric parameters at week 16**			
**Weight (Kg, SD)**	44 ± 9.9	43.3 ± 12.6	0.86
**Height (cm, SD)**	155.7 ± 16.4	153.1 ± 15.9	0.66
**BMI (Kg/m², SD)**	17.9 ± 1.3	18 ± 2.7	0.86

[2S] Anthropometric parameters in both groups recorded at week 0, 8 and 16.
